# Evolution of Bluetooth Technology: BLE in the IoT Ecosystem

**DOI:** 10.3390/s25040996

**Published:** 2025-02-07

**Authors:** Grigorios Koulouras, Stylianos Katsoulis, Fotios Zantalis

**Affiliations:** TelSiP Research Laboratory, Department of Electrical and Electronic Engineering, School of Engineering, University of West Attica, Ancient Olive-Grove Campus, GR-12241 Athens, Greece; skatsoulis@uniwa.gr (S.K.); fzantalis@uniwa.gr (F.Z.)

**Keywords:** Bluetooth Low Energy (BLE), Internet of Things (IoT), IoT connectivity, low-power wireless communication, BLE mesh networking, BLE security and privacy, industrial IoT (IIoT), future trends in BLE

## Abstract

The Internet of Things (IoT) has witnessed significant growth in recent years, with Bluetooth Low Energy (BLE) emerging as a key enabler of low-power, low-cost wireless connectivity. This review article provides an overview of the evolution of Bluetooth technology, focusing on the role of BLE in the IoT ecosystem. It examines the current state of BLE, including its applications, challenges, limitations, and recent advancements in areas such as security, power management, and mesh networking. The recent release of Bluetooth Low Energy version 6.0 by the Bluetooth Special Interest Group (SIG) highlights the technology’s ongoing evolution and growing importance within the IoT. However, this rapid development highlights a gap in the current literature, a lack of comprehensive, up-to-date reviews that fully capture the contemporary landscape of BLE in IoT applications. This paper analyzes the emerging trends and future directions for BLE, including the integration of artificial intelligence, machine learning, and audio capabilities. The analysis also considers the alignment of BLE features with the United Nations’ Sustainable Development Goals (SDGs), particularly energy efficiency, sustainable cities, and climate action. By examining the development and deployment of BLE technology, this article aims to provide insights into the opportunities and challenges associated with its adoption in various IoT applications, from smart homes and cities to industrial automation and healthcare. This review highlights the significance of the evolution of BLE in shaping the future of wireless communication and IoT, and provides a foundation for further research and innovation in this field.

## 1. Introduction

Bluetooth has revolutionized wireless communication by replacing cumbersome wired connections, transforming how devices interact. Since its inception in the late 1990s, Bluetooth has continuously evolved, becoming a versatile standard that facilitates seamless connectivity and efficient data transfer. This manuscript addresses the evolution of Bluetooth and its influence on the future of wireless communication. While the development of Bluetooth involved a team effort, the credit for inventing the core technology goes to Jacobus Cornelis Haartsen, a Dutch electrical engineer working at Ericsson Mobile. In the late 1990s, Bluetooth’s journey began with the version 1.0, by replacing clunky cables with short-range wireless connections for headsets and phones. The early 2000s saw a surge in Bluetooth adoption with features like data transfer. The introduction of Bluetooth Low Energy (BLE) in 2010 marked a significant milestone in Bluetooth technology. Gomez et al. provided an early overview and evaluation of BLE, highlighting its potential as an emerging low-power wireless technology [1]. This early assessment proved prescient, as BLE went on to revolutionize the landscape with its ultra-low power consumption, paving the way for the widespread use of Bluetooth in wearables, smart homes, and the ever-growing Internet of Things (IoT). Bluetooth Technology utilizes short-range radio waves within the 2.4 GHz Industrial, Scientific, and Medical (ISM) band to connect devices wirelessly. This wireless personal area network technology, overseen by the Bluetooth Special Interest Group (Bluetooth SIG), comes in two main types: Bluetooth Classic and Bluetooth Low Energy. Bluetooth Classic or Bluetooth Basic Rate/Enhanced Data Rate (BR/EDR) is described in Bluetooth Core Specification versions 1.0 to 3.0. A major shift came in 2010 with Bluetooth version 4.0, introducing Bluetooth Low Energy. This entirely new protocol operates largely independently of Bluetooth Classic. Compared to Bluetooth Classic, BLE is intended to provide considerably reduced power consumption and cost while maintaining a similar communication range. BLE is independent of BR/EDR and has no compatibility, but they are able to coexist. Quite early, as of 2017, Mikhaylov et al. [2] conducted a comparative analysis of BLE, IEEE 802.15.4, and SimpliciTI, demonstrating the superior performance of BLE in energy efficiency, low latency, and theoretical throughput, which solidifies its position as a leading technology for IoT applications. The theoretical maximum throughput of BLE of 320 kbps and a frame turnaround time of under 1 ms highlight its responsiveness and suitability for low-power scenarios. While real-world implementations face software limitations that reduce throughput, with BLE, the significant energy savings and optimized design for short-range communication make it ideal for IoT devices requiring extended battery life and reliable performance. This analysis emphasizes the practicality of BLE and its advantage in supporting modern IoT ecosystems.

At its core, the IoT ecosystem refers to the interconnected network of physical devices, sensors, software, and networks that collect and exchange data to create smarter and more responsive systems. In an IoT ecosystem, devices can include anything from wearable health monitors and smart home appliances to industrial machines and connected vehicles. These devices are typically equipped with sensors that track parameters such as temperature, motion, or location and actuators that enable devices to perform tasks in response to the collected data. Connectivity, via wireless protocols such as BLE, Wi-Fi, or cellular, allows these devices to communicate with each other or transmit data to edge or cloud platforms for analysis. These data may then trigger automated actions, inform human decision-making, or be visualized in dashboards. Security is essential throughout this process, safeguarding both device integrity and data privacy. By uniting devices, networks, and intelligent data processing, an IoT ecosystem can bring about efficiencies, improve user experiences, and even enable entirely new business models across diverse sectors.

The primary purpose of this survey is to provide a comprehensive review of the evolution of Bluetooth technology, with a particular focus on BLE, and its pivotal role in the IoT ecosystem. This involves tracing the progression of Bluetooth technology from its inception to its current state, highlighting key milestones and technological advancements. It aims to offer a detailed analysis of the technical specifications, features, and enhancements of BLE over traditional Bluetooth technology. Additionally, it identifies the technical challenges associated with the implementation of BLE in IoT and explores the solutions developed to address these issues. Lastly, the survey discusses emerging trends and potential future advancements in BLE technology and its impact on IoT. Darroudi and Gomez provided an early survey of BLE mesh networks, a technology that has since become crucial for large-scale IoT deployments [3]. More recently, Natgunanathan et al. have explored the current state-of-the-art in BLE mesh, its applications, and considerations for implementation [4]. Understanding the evolution of BLE in the IoT ecosystem is crucial for several reasons. It provides insights into how wireless communication technologies have progressed, leading to more efficient, low-power solutions essential for IoT devices. The role of BLE in IoT highlights its significance in enabling seamless, reliable, and energy efficient connectivity among a wide array of devices, from wearable technology to smart home systems. A comprehensive survey serves as a valuable resource for researchers, developers, and industry professionals by providing a consolidated knowledge base, thus facilitating informed decision-making and strategic planning. Understanding the current trends and potential future directions of BLE and IoT helps in anticipating future market demands and technological shifts, ensuring that current and upcoming solutions remain relevant and effective. The insights gained from this survey can benefit multiple disciplines beyond IoT, such as healthcare, automotive, and industrial automation, where BLE technology is increasingly being adopted. While BLE has become a dominant technology in short-range, low-power communication, it is important to consider it within the broader ecosystem of IoT-enabling technologies. Raza et al. [5] provided an overview of Low-Power Wide Area Networks (LPWANs), which complement BLE in scenarios requiring longer-range communication. As BLE continues to evolve and integrate more deeply into IoT ecosystems, new challenges will emerge, particularly in the areas of security and privacy. Cui et al. [6] highlighted these challenges in the context of smart cities, an area where BLE-enabled IoT devices are becoming increasingly prevalent. Addressing these security and privacy concerns will be crucial for the continued growth and acceptance of BLE in critical IoT applications.

This review employs a systematic approach to the literature selection. Research was sourced from peer-reviewed journals, recognized conference proceedings, and reputable industry white papers, focusing on the historical development of Bluetooth technology with an emphasis on BLE versions, technical advancements and performance metrics, application scenarios in various IoT domains, and the associated challenges along with emerging trends. Inclusion criteria emphasized recent works (2015–2024), addressing the technical evolution of BLE and IoT integration. Works that offered substantive technical insights (either historical or contemporary) on the BLE specifications, performance characteristics, security features, or real-world IoT applications were included. Foundational or early works that, although older, provided essential context to understand the emergence and progression of BLE were also considered. Exclusion criteria omitted most non-peer-reviewed sources and outdated studies, ensuring reliability and relevance. Comparative analysis with competing wireless technologies, such as Zigbee, LoRa, and Wi-Fi, was considered out of scope for this article, and therefore, these topics were omitted. Sources that did not meet basic academic or technical rigor, such as brief editorials or extended abstracts without data, were excluded. Finally, the review focused on English-language works to ensure the accuracy of the synthesis and maintain consistency throughout.

The paper begins with an introduction that provides the background on Bluetooth technology, outlines the purpose of this survey, and explains its importance in understanding the evolution of BLE within the IoT ecosystem. The introduction also includes a description of the methodology used to conduct the review, which details the approach taken for literature selection and analysis. Following the introduction, the paper presents an overview of Bluetooth technology, tracing its historical development and highlighting key advancements across different versions. The Section 2 delves into the evolution of Bluetooth Low Energy, detailing its unique characteristics, technical specifications, and development phases. Subsequently, the paper discusses the integration of BLE into the IoT ecosystem, discussing its role, specific applications, and case studies. This section also explores the role of BLE technology in supporting the achievement of the United Nations’ Sustainable Development Goals. This is followed by an examination of the technical challenges associated with implementing BLE in IoT and the solutions developed to address these challenges. The paper then discusses future trends and potential advancements in BLE technology and its implications for IoT. Finally, the conclusion summarizes the key points, emphasizes the significance of the evolution of BLE in the context of wireless communication and IoT, and offers final thoughts on its ongoing development. The paper is rounded off with a comprehensive list of references, citing all the academic papers, articles, and sources used in the survey.

## 2. Overview of Bluetooth Technology

The evolution of Bluetooth technology has significantly influenced the landscape of wireless communication. Since its inception in the 1990s, Bluetooth has evolved from simple cable replacement technology to a sophisticated and versatile wireless standard that supports a wide range of applications. This section provides a comprehensive overview of Bluetooth technology, tracing its historical development, key milestones, and the critical advancements that have propelled it to its current state. By understanding the foundational aspects and the progressive enhancements of Bluetooth, we can appreciate its role in modern wireless communication and its transformative impact on various industries, particularly the Internet of Things ecosystem.

### 2.1. Historical Perspective: Development and Milestones in Bluetooth Technology

**Figure 1:**
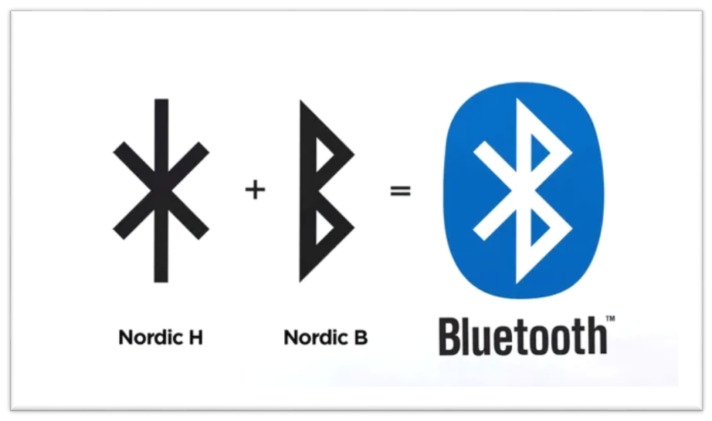
Superimposition of the Nordic runes for the letters H and B, representing “Harald Bluetooth”.

Bluetooth technology has undergone significant evolution since its inception, marked by several key milestones that have shaped its development and widespread adoption. Bluetooth was conceptualized in the mid-1990s by Ericsson engineers, with the aim of creating a wireless alternative to RS-232 data cables. The name “Bluetooth” was inspired by the 10th century Scandinavian king Harald “Bluetooth” Gormsson, who united Denmark and Norway, symbolizing the intent of the technology to unite different devices [7]. The superimposition of the Nordic runes for the letters H and B, representing “Harald Bluetooth”, is shown in Figure 1.

The first version of Bluetooth, 1.0, was released in 1999 [8], followed by version 1.1 in 2001 [9] and 1.2 in 2003 [10]. These versions provided basic wireless connectivity with a data transfer rate of up to 721 kbps but faced issues with interoperability and complexity in pairing devices. In 2004, Bluetooth 2.0 + Enhanced Data Rate (EDR) was introduced [11], followed by Bluetooth 2.1 in 2007 [12], both of which increased the data transfer rate to 3 Mbps and lowered power consumption. These advancements greatly improved the user experience by making the technology more dependable and efficient. Bluetooth 3.0 + High Speed (HS), released in 2009 [13], leveraged Wi-Fi technology to achieve data transfer rates up to 24 Mbps. This version was designed for applications requiring high-speed data transfer, such as file sharing and media streaming. The introduction of Bluetooth 4.0 in 2010 [14] marked a significant milestone with the introduction of Bluetooth Low Energy. BLE was designed for applications requiring low power consumption, enabling devices to operate for longer periods on smaller batteries. This version expanded the scope of Bluetooth technology, making it suitable for the emerging Internet of Things market. Bluetooth 4.1, released in 2013 [15], brought improvements in coexistence with LTE networks, better connections, and enhanced data transfer efficiency. Bluetooth 4.2, introduced in 2014 [16], further enhanced security features and increased the data packet capacity, which facilitated faster and more reliable communication. Bluetooth 5.0, launched in 2016 [17], offered significant enhancements, including four times the range, twice the speed, and eight times the data broadcasting capacity of BLE. This version was optimized for IoT applications, providing better coverage and higher throughput, which are critical for smart homes, industrial IoT, and other large-scale deployments. Bluetooth 5.1, released in 2019 [18], introduced features like direction finding, which improved location services by providing centimeter-level accuracy. Bluetooth 5.2, introduced on 31 December 2019 [19], brought enhancements in audio streaming with the introduction of Low-Energy Audio (LE Audio), improved power efficiency, and increased data transmission efficiency. Bluetooth 5.3, released in 2021 [20], introduced improvements in connection subrating, periodic advertising enhancement, and encryption key size control, enhancing overall performance and security. Bluetooth 5.4, launched in 2023 [21], featured advancements in Periodic Advertising with Responses (PAwR), enabling more efficient data transfers and improved location services. The most recent version, Bluetooth 6.0, which adopted by the Bluetooth SIG Board of Directors in 2024 [22], promises significant enhancements in speed, range, and reliability, with a particular focus on supporting advanced audio applications and IoT devices. Each iteration of Bluetooth technology has built upon the previous versions, enhancing data rates, power efficiency, security, and overall performance. These advancements have expanded Bluetooth’s applicability from simple cable replacement to being a key enabler of modern wireless communication, particularly in the rapidly growing IoT ecosystem. The historical development of Bluetooth technology highlights its evolution as a versatile and ubiquitous connectivity solution, continually adapting to meet the changing needs of consumers and industries.

### 2.2. Key Features and Advancements in Different Versions of Bluetooth

Bluetooth technology has evolved through several versions, as depicted in Table 1, each introducing key features and advancements that have enhanced its functionality, performance, and applicability. The initial versions, Bluetooth 1.0 [8], 1.1 [9] and 1.2 [10], provided basic wireless connectivity but were hampered by interoperability issues and complex pairing processes. Bluetooth 2.0 + Enhanced Data Rate (EDR) [11] and 2.1 [12] significantly improved data transfer rates and power efficiency, making the technology more reliable and user-friendly. The introduction of Bluetooth 3.0 + High Speed (HS) [13] leveraged Wi-Fi technology to achieve higher data transfer speeds, catering to applications requiring rapid data exchange. Bluetooth 4.0 [14] marked a significant milestone with the introduction of Bluetooth Low Energy, designed for low power consumption and extended battery life, which expanded its suitability for the emerging IoT market. Subsequent versions, such as Bluetooth 4.1 [15] and 4.2 [16], brought enhancements in coexistence with LTE networks, increased data capacity, and improved security features. Bluetooth 5.0 [17] further revolutionized the technology by increasing range, speed, and data broadcasting capacity, optimizing it for IoT applications. The latest versions, including Bluetooth 5.1 [18], 5.2 [19], 5.3 [20], 5.4 [21], and 6.0 [22], introduced advanced features such as direction finding, LE Audio for enhanced audio streaming, and further optimizations in speed, range, power efficiency, and connection reliability. These continuous advancements have solidified Bluetooth’s position as a versatile and essential wireless communication standard.

### 2.3. Bluetooth Device Classes: Power and Range Specifications

Bluetooth technology categorizes its devices into different classes based on their power output and intended range of operation [22]. These classes define the maximum allowable transmission power and consequently influence the communication range. Understanding these classes is essential for selecting the appropriate Bluetooth devices for specific applications, ensuring optimal performance and power efficiency. Class 1 Bluetooth devices are designed for long-range communication, typically offering a range of up to 100 m (328 feet) or more. These devices operate at a maximum transmission power of 100 mW or 20 dBm. Due to their higher power output, Class 1 devices are commonly used in industrial applications, outdoor environments, and situations where extended range and robust connectivity are required. Examples include industrial automation systems, wireless sensors in large facilities, and some outdoor wireless audio systems. Class 1.5 Bluetooth devices represent an intermediate power class, offering a balance between the extended range of Class 1 and the power efficiency of Class 2. These devices operate at a maximum transmission power of 10 mW or 10 dBm, typically providing a range of up to 30 m (98 feet). Class 1.5 is particularly useful in scenarios where a longer range than Class 2 is needed, but the full power and range of Class 1 are unnecessary or impractical due to power constraints. This class is often employed in smart home devices, wearable technology, and certain IoT applications where moderate range and optimized power consumption are crucial. Examples include smart locks, fitness trackers, and some wireless audio devices that require coverage throughout a typical home or small office environment. Class 2 Bluetooth devices are the most common type found in consumer electronics, including smartphones, tablets, laptops, and other portable devices. They offer a moderate range of up to 10 m (33 feet) and operate at a maximum transmission power of 2.5 mW or 4 dBm. This class strikes a balance between power consumption and communication range, making it suitable for most personal and office use cases. Class 2 devices are typically used for wireless headsets, keyboards, mice, and other peripheral devices that connect to personal computing devices. Class 3 Bluetooth devices are designed for short-range communication, typically offering a range of up to 1 m (3 feet). These devices operate at a maximum transmission power of 1 mW or 0 dBm. Due to their limited range, Class 3 devices are less common and are generally used in specialized applications where close-proximity communication is sufficient. Examples include some medical devices, Personal Area Networks (PANs), and short-range data transfer applications. While not commonly mentioned, Class 4 Bluetooth devices are a theoretical category that would operate at an even lower transmission power than Class 3, with a correspondingly shorter range. These devices would be used in ultra-low power, very short-range applications where conserving battery life is paramount, and the communication range is very limited. The classification of Bluetooth devices into different classes based on their power output and range capabilities allows for a wide variety of applications, from short-range personal devices to long-range industrial systems. Class 1 devices cater to applications requiring extended range and robust connectivity, Class 2 devices are ideal for most consumer electronics, and Class 3 devices serve niche applications requiring close-proximity communication. Understanding these classes helps in selecting the right Bluetooth device for specific use cases, ensuring optimal performance and efficiency. Table 2 provides a comparison of the different classes of Bluetooth devices, highlighting their maximum transmission power, typical range, and usage scenarios for both BLE and Bluetooth Classic Basic Rate/Enhanced Data Rate (BR/EDR).

## 3. Evolution of Bluetooth Low Energy

### 3.1. Key Characteristics of BLE and Differences from Bluetooth Classic

Bluetooth Low Energy was introduced as part of the Bluetooth 4.0 specification in 2010, representing a significant advancement in wireless communication technology. Unlike Bluetooth Classic, which was designed for continuous, high-bandwidth data streaming applications, BLE focuses on providing a low-power solution for devices that require periodic data transfer. One of the key characteristics of BLE is its ability to operate with significantly reduced power consumption, enabling devices to run on small batteries for extended periods, sometimes for several years, without the need for frequent recharging or replacement. A plethora of researchers highlight the significant advantage of BLE in power efficiency, emphasizing its design for low-energy applications, which is crucial for devices that require prolonged battery life in various IoT contexts [23,24,25]. BLE achieves this low power consumption by using short, intermittent transmission bursts instead of continuous streaming. This makes it ideal for applications in IoT, where devices often need to send small amounts of data infrequently. Additionally, BLE features a simplified protocol stack compared to classic Bluetooth, which reduces complexity and further conserves power. Another critical differentiator is the scalability and flexibility of BLE in supporting a wide range of devices and applications, from simple sensors and beacons to more complex devices like fitness trackers and medical monitors. BLE also offers robust security features, including AES-128 encryption, to ensure that data transmitted over BLE connections are secure. Çetintav and Taşkın in their study discuss the application of the Advanced Encryption Standard (AES) [26] algorithm in BLE modules, emphasizing its effectiveness in securing data generated on IoT devices. It highlights that despite being resource intensive, AES can protect data effectively in BLE environments [27]. These characteristics make BLE a versatile and essential technology in modern wireless communication, particularly within the IoT ecosystem, where efficiency, power conservation, and secure data transfer are paramount.

### 3.2. Technical Specifications: Detailed Technical Aspects of BLE

To fully understand the capabilities and limitations of BLE in IoT applications, it is essential to delve into its technical specifications.

This subsection provides an in-depth examination of the technical aspects of BLE, including its power consumption, range, throughput, latency, advertising and scanning, security, topology, frequency, and modulation. The key technical specifications of BLE, compared to its predecessor Bluetooth Classic, are summarized in Table 3, highlighting differences in channels, throughput, radio profiles, power consumption, range, and network topologies.

#### 3.2.1. Power Consumption

One of the most notable features of BLE is its remarkably low power consumption. BLE achieves this by using short bursts of data transmission and extended periods of inactivity, known as “nap”, “sleep” or “deep sleep” states. This allows devices to operate on small batteries for extended periods, often up to several years, depending on the usage pattern. The low duty cycle, combined with efficient use of the radio, significantly reduces power draw compared to Bluetooth Classic. Bluetooth technology supports transmit powers from −20 dBm (0.01 mW) to +20 dBm (100 mW).

#### 3.2.2. Range

BLE offers a versatile range that can be finely tuned according to the specific requirements of an application. Typically, in open environments characterized by Line-of-Sight (LOS) and minimal radio frequency interference, BLE devices can achieve a range of up to 100 m. However, this range is highly susceptible to environmental factors: (a) obstacles (e.g., thick walls and metal barriers) can significantly reduce the effective range, and (b) interference (e.g., from Wi-Fi routers and microwaves) may degrade the signal quality. To extend the range, BLE devices can utilize higher transmit power settings, albeit at the cost of increased power consumption.

Moreover, a significant enhancement to the range capabilities of BLE was introduced with BLE in Long Range mode, also known as LE Coded PHY [17,18,19,20,21,22]. This feature is specifically designed to enhance the range of Bluetooth Low Energy communication without necessitating an increase in transmit power, thereby maintaining power efficiency. LE Coded PHY achieves this through Forward Error Correction (FEC) and coding schemes that allow for more reliable data transmission over longer distances. The implementation of LE Coded PHY has a profound impact on the achievable range of BLE devices. In theoretical terms, it can extend the range up to four (4) times the original distance, reaching approximately 400 m under ideal conditions. More practically, this enhancement significantly improves connectivity in challenging environments, which particularly benefits applications that rely on reliable long-range communication, such as those found in industrial or smart infrastructure settings. Additionally, the sensitivity of the receiver remains a critical factor in maintaining reliable long-range communication. The wireless signal degrades predictably over distance, following the inverse square law. For example, a low-sensitivity receiver, with a sensitivity below −90 dBm, may struggle to decipher information accurately at longer ranges. With a 6 dBm sensitivity increase, the range should theoretically double. Advancements in receiver technology, such as improved filtering and amplifier designs, have further enhanced the sensitivity of modern BLE devices, thereby supporting more robust long-range communication.

#### 3.2.3. Throughput

The data rate of BLE is designed to balance power efficiency with sufficient bandwidth for a wide range of applications. BLE supports data rates of 125 kbps, 500 kbps, and 1 Mbps, with the possibility of achieving up to 2 Mbps using the LE 2M PHY feature introduced in Bluetooth 5.0, as can be seen in Table 3. While these rates are lower than those of Bluetooth Classic, they are adequate for small, periodic data transmissions typical in IoT applications. The mandatory physical layer (PHY) in Bluetooth 4.0 is the LE 1M PHY, which utilizes Gaussian Frequency Shift Keying (GFSK) modulation with a symbol rate and a bit rate of 1 Msym/s and 1 Mbps, respectively. Bluetooth 5.0 introduces two optional PHYs: the LE 2M PHY, which doubles the speed with a symbol rate of 2 Msym/s and a bit rate of 2 Mbps using two-level GFSK, and the LE Coded PHY, which enhances range through Forward Error Correction coding. The LE Coded PHY supports two schemes: S = 2, which achieves a bit rate of 500 Kbps and doubles the range, and S = 8, which achieves a bit rate of 125 Kbps and quadruples the range, without increasing the transmission power [28].

#### 3.2.4. Latency

BLE is optimized for low-latency communication, which is crucial for real-time applications. The connection latency in BLE can be as low as a few milliseconds, depending on the connection interval settings. This ensures timely data exchange and responsiveness, which is essential for applications like health monitoring and fitness tracking.

#### 3.2.5. Advertising and Scanning

BLE uses an advertising model to establish connections and broadcast data. Devices can send out advertisement packets at configurable intervals, which can be received by scanning devices. This model allows for an efficient discovery and connection setup, making it ideal for applications like location-based services and proximity sensing.

#### 3.2.6. Security

Security in BLE is robust, with support for AES-128 encryption to protect data transmitted between devices. BLE also implements secure pairing mechanisms, such as Passkey Entry, Just Works, and Out-of-Band (OOB) pairing, to ensure that connections are established securely and data integrity is maintained. Bluetooth 6.0 introduces significant enhancements in privacy and security through Resolvable Private Addresses (RPAs) and a new physical layer configuration known as LE 2M 2BT [22]. RPAs work by periodically changing device addresses, making it difficult for third parties to track devices using static identifiers like MAC addresses. The use of an Identity Resolving Key (IRK) ensures that only authorized devices can associate the RPA with a known identity, enhancing privacy and anonymity while mitigating security threats related to persistent device tracking. Additionally, LE 2M 2BT introduces Channel Sounding for secure and accurate distance measurements, which is particularly useful in environments requiring precise positioning, such as augmented reality applications. The Bandwidth-Bit Period Product (BT) parameter influences signal pulse characteristics, with a BT value of 2.0 enhancing security by making channel sounding resistant to physical layer attacks. This ensures that wireless communications are secure from interception attempts. In essence, Bluetooth 6.0’s features address privacy through dynamic addresses and enhance security through improved distance measurements and robust physical layer encryption, providing a comprehensive approach to user privacy and device protection.

#### 3.2.7. Topology

BLE supports various topologies, including Point-to-Point (P2P), broadcast, and mesh networking. P2P is used for direct communication between two devices, while broadcasting allows one device to send data to multiple devices without establishing a connection. The mesh-based model hierarchy introduced in Bluetooth 5.1 [18] enables many-to-many communication, an essential capability for complex IoT systems like smart lighting and industrial automation.

#### 3.2.8. Frequency and Modulation

**Figure 2:**
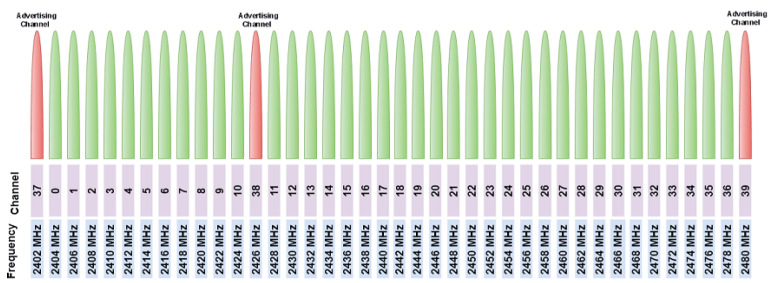
Mapping between the frequencies and Bluetooth LE channels. (Source: https://es.mathworks.com/help/bluetooth/ug/bluetooth-low-energy-waveform-generation-and-visualization.html (Mathworks) (accessed on 3 February 2025)).

The Bluetooth LE system operates in the 2.4 GHz ISM band at 2400–2483.5 MHz, using 40 channels, each 2 MHz wide. Figure 2 shows the mapping between the frequencies and Bluetooth LE channels. Each of these RF channels is allocated a unique channel index. It employs GFSK modulation, which provides robust communication with low power consumption. The use of the Frequency Hopping Spread Spectrum (FHSS) helps mitigate interference and improve communication reliability.

#### 3.2.9. Summarize

These technical specifications highlight the ability of BLE to deliver efficient, low-power, and reliable wireless communication, making it an ideal choice for a wide range of IoT applications.

### 3.3. Development Phases: Evolution Stages of BLE from Its Inception to the Latest Updates

**Figure 3:**
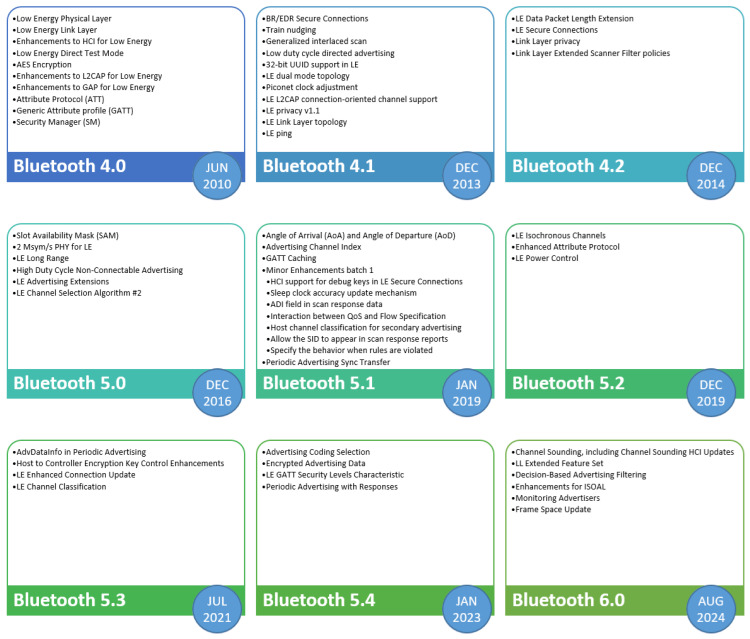
Evolution of BLE technology.

The development of BLE has been marked by several significant phases, each introducing improvements and new features that have broadened its applications and improved its performance. Table 4 and Figure 3 depict the key stages in the evolution of BLE from its inception to the most recent updates.

BLE was officially introduced with the Bluetooth 4.0 specification in 2010 [14]. This initial version of BLE was designed to provide low power consumption while maintaining effective communication over short distances. Key features included a simplified protocol stack, efficient data transmission methods, and enhanced security. The low power profile of BLE made it ideal for applications such as fitness trackers, medical devices, and other battery-operated gadgets. Bluetooth 4.1 [15], released in 2013, brought several improvements to BLE. This version focused on better coexistence with LTE networks, enhancing the reliability of BLE in environments with cellular interference. Bluetooth 4.1 also introduced features that allowed devices to operate simultaneously as a peripheral and a central, improving flexibility and interoperability in complex IoT setups. Additionally, it provided developers with more control over the connection parameters, allowing for optimized performance based on specific application needs. The release of Bluetooth 4.2 [16] in 2014 marked another significant milestone in the evolution of BLE. This version enhanced privacy and security features, making it more robust against unauthorized access and tracking. Bluetooth 4.2 also increased the data packet capacity, which facilitated faster and more efficient data transfers. The introduction of Internet Protocol Support Profile (IPSP) enabled BLE devices to connect directly to the internet, expanding their potential applications in the IoT ecosystem.

Bluetooth 5.0 [17], launched in 2016, introduced major performance enhancements to BLE. It offered four times the range, twice the speed, and eight times the data broadcasting capacity compared to previous versions. These improvements made BLE more suitable for large-scale IoT deployments, such as smart homes, industrial automation, and outdoor sensor networks. Bluetooth 5.0 also introduced new features like LE 2M PHY, which allowed for higher data rates, and LE Coded PHY, which extended range without compromising data integrity. Bluetooth 5.1 [18], released in 2019, brought significant advancements in location services. The introduction of direction finding enabled BLE devices to determine the direction of a signal, providing centimeter-level accuracy for indoor positioning and navigation applications. Bluetooth 5.2 [19] introduced at the end of 2019, prioritized enhancing audio streaming with the debut of LE Audio. This included features such as the Low-Complexity Communication Codec (LC3) for higher audio quality at lower bit rates and multi-stream audio support, which enhanced the performance of wireless audio devices. Bluetooth 5.3 [20], released in 2021, continued to refine the performance and efficiency of BLE. This version introduced features aimed at further reducing power consumption and improving connection reliability. Enhanced periodic advertising allowed for more efficient use of broadcasting channels, which is particularly beneficial for applications requiring regular updates, such as environmental monitoring and asset tracking. Bluetooth 5.3 also included improvements in channel selection algorithms, which helped mitigate interference and enhance overall communication stability. Bluetooth 5.4 [21], released in 2023, brought further enhancements tailored for the growing demands of IoT. This version introduced Periodic Advertising with Responses (PAwR), which allowed devices to exchange data more efficiently in a synchronized manner, reducing latency and improving power efficiency. Bluetooth 5.4 also enhanced the support for Electronic Shelf Labels (ESLs), making it more suitable for large-scale retail applications where numerous devices need to communicate simultaneously and reliably. Additionally, improvements in the robustness and security of connections ensured that BLE continued to meet the stringent requirements of modern IoT applications. The latest Bluetooth iteration, version 6.0 [22], was launched in August 2024, revolutionizing the technology with innovative features and improvements that aim to elevate the developer experience and reshape user interactions across multiple platforms. With its release, Bluetooth 6.0 introduced significant upgrades that boost performance, efficiency, and adaptability, paving the way for a new wave of applications and use cases. Key innovations include “Bluetooth^®^ Channel Sounding”, which introduces true distance awareness for precise location tracking, improving solutions like “Find My” and adding robust security for digital keys by ensuring only authorized users within range can gain access. “Decision-Based Advertising Filtering” optimizes Bluetooth LE scanning by allowing devices to filter packets more effectively, reducing unnecessary energy consumption. The “Monitoring Advertisers” feature improves connection reliability by notifying devices when those of interest move in or out of range, preventing wasted scanning efforts. Enhancements to the “Isochronous Adaptation Layer (ISOAL)” reduce latency in time-sensitive applications, while the “LL Extended Feature Set” enables devices to communicate more advanced capabilities, ensuring better interoperability for complex use cases. Finally, the negotiable “Frame Space Update” allows devices to customize transmission intervals, improving performance for applications requiring low latency or high data efficiency. These updates position Bluetooth 6.0 as a cornerstone for next-generation IoT, smart devices, and connected ecosystems. Each iteration of Bluetooth technology has built upon the previous versions, enhancing data rates, power efficiency, security, and overall performance. These advancements have expanded Bluetooth’s applicability from simple cable replacement to a key enabler of modern wireless communication, particularly in the rapidly growing IoT ecosystem [29]. The historical development of Bluetooth technology highlights its evolution as a versatile and ubiquitous connectivity solution, continually adapting to meet the changing needs of consumers and industries.

### 3.4. BLE in Long Range Mode

The Bluetooth in Long Range mode, also known as LE Coded PHY, is a feature introduced with Bluetooth 5.0 that enhances the range of Bluetooth Low Energy communication [17]. This technology aims to improve the reliability and distance over which BLE devices can communicate, making it more suitable for a wider range of applications, particularly in the Internet of Things. BLE Long Range significantly extends the communication range of BLE devices, up to four times compared to previous versions. This means it can achieve distances well beyond the typical 100 m (328 feet) seen in standard BLE, reaching up to 400 m (1312 feet) or more in optimal conditions [28]. The key to BLE Long Range is the LE Coded PHY (Physical Layer), which uses FEC to enhance data robustness and reliability. There are two coding schemes available:

- S = 2: Provides a data rate of 500 kbps with increased range.
- S = 8: Provides a data rate of 125 kbps with maximum range.

The incorporation of FEC reduces the impact of interference and noise, improving the reliability of the connection over longer distances. This is particularly beneficial in environments plagued by RF interference or physical obstructions.

Notably, BLE Long Range maintains low power consumption, making it suitable for battery-operated devices. It operates within the same 2.4 GHz ISM band as standard BLE, utilizing GFSK modulation with added coding schemes for Forward Error Correction. However, it is important to note that the use of these advanced coding schemes and the need to transmit data over a longer period to achieve extended range result in slightly increased power consumption compared to uncoded BLE schemes. Despite this trade-off, the overall power consumption remains low, particularly in IoT applications where continuous data transmission is not required. This makes BLE Long Range an efficient choice for such use cases. The backward compatibility with previous BLE versions further solidifies its position as a versatile solution, ensuring seamless integration with existing IoT infrastructures.

#### Real-World Performances

Several real-world experiments have demonstrated the capabilities of Bluetooth Low Energy technology in achieving long-range communication. For instance, in January 2017, two engineers of Texas Instruments, Espen Wium and Fredrik Georg Kervel successfully conducted an outdoor experiment on the hills surrounding Oslo to illustrate the capabilities of their SimpleLink Bluetooth low energy CC2640R2F wireless microcontroller. Their study effectively showcased how this device can utilize Bluetooth long-range modes, also known as coded PHYs, achieving a notable range of over 1500 m with a transmit power level of +5 dbm [30]. Other companies have carried out similar experiments, including Nordic Semiconductor, which in May 2018 successfully tested the effectiveness of BLE Long Range using their nRF52840 SoC chip [31]. In their experiment, titled “Testing Long Range Coded PHY with Nordic’s solution—it simply works”, the results showed that BLE signals with LE Coded PHY and 0 dbm transmit power could be received at a distance of up to 1300 m. STMicroelectronics has also demonstrated the long-range capabilities of BLE, achieving distances exceeding 1600 m with their STM32WBA series [32]. These experiments highlight the potential of BLE technology to support extended range applications through specialized designs and implementations.

## 4. BLE in the IoT Ecosystem

### 4.1. IoT Overview: Explanation of the IoT Ecosystem and Its Components

The Internet of Things (IoT) refers to the interconnected network of physical devices, vehicles, buildings, and other objects embedded with sensors, software, and network connectivity that enable them to collect and exchange data [33]. This ecosystem is designed to create a seamless integration between the physical and digital worlds, enhancing the efficiency, productivity, and convenience of various applications across different industries. At the core of the IoT ecosystem are the “devices” themselves, often referred to as “smart” devices [34]. These include everyday objects like home appliances, wearable technology, industrial machinery, and even entire infrastructures such as smart cities. Each device is equipped with sensors that monitor various parameters, such as temperature, humidity, motion, and location, and actuators that allow devices to perform specific actions in response to the data received. “Connectivity” is another critical component of the IoT ecosystem [35]. Devices use various communication protocols to connect and share data with each other and central systems. These protocols include Wi-Fi, cellular networks, Zigbee, Z-Wave, and particularly BLE, which offers low power consumption and effective communication over short distances. The “data” generated by IoT devices are collected and transmitted to centralized systems or cloud platforms for analysis and processing. These platforms employ advanced analytics and machine learning algorithms to extract meaningful insights from the raw data, enabling predictive maintenance, operational optimization, and enhanced decision-making processes. “Edge computing” is a growing trend within the IoT ecosystem, where data processing occurs closer to the data source, at the edge of the network, rather than relying solely on centralized cloud services [36]. This approach reduces latency, improves response times, and alleviates the bandwidth load on central systems, making it particularly useful for real-time applications. “Security” and “privacy” are paramount in the IoT ecosystem [37]. As more devices become interconnected, the potential for cyber threats increases. Ensuring the confidentiality, integrity, and availability of data is crucial. This involves implementing robust encryption methods, secure communication protocols, and access control mechanisms to protect against unauthorized access and data breaches. The IoT ecosystem is also supported by various standards and regulations that ensure interoperability, reliability, and safety of devices and systems. Organizations like the International Organization for Standardization (ISO) [38], the Institute of Electrical and Electronics Engineers Standards Association (IEEE SA) [39], and the Internet Engineering Task Force (IETF) [40] develop and maintain these standards. In essence, the IoT ecosystem comprises a vast network of connected devices, reliable communication protocols, robust data analytics, and stringent security measures. It transforms how we interact with the physical world, leading to smarter homes, efficient industries, improved healthcare, and enhanced environmental monitoring, among many other applications. Bluetooth Low Energy plays a crucial role in this ecosystem by providing the necessary connectivity and power efficiency to support a wide range of IoT devices and applications.

### 4.2. Integration of BLE in IoT: How BLE Fits into and Enhances the IoT Framework

Bluetooth Low Energy is an integral component of the IoT framework, providing the necessary connectivity, efficiency, and flexibility to support a wide range of IoT applications. The low power consumption of BLE is one of its most significant advantages, allowing devices to operate on small batteries for extended periods, which is essential for IoT devices that are often deployed in remote or difficult-to-access locations. This energy efficiency makes BLE ideal for battery-powered sensors, wearable devices, and other applications where frequent recharging or battery replacement is impractical. The capability of BLE for short-range communication fits well with many IoT use cases, such as smart homes, healthcare, and industrial automation. In a smart home, BLE can connect various devices like thermostats, lighting systems, and security cameras, enabling seamless control and automation. For healthcare applications, BLE is used in wearable health monitors and medical devices, allowing for continuous patient monitoring and data collection without the need for frequent battery replacements. The simplicity of the BLE protocol stack reduces complexity and cost in device implementation, facilitating the deployment of a large number of interconnected devices. This streamlined communication protocol supports the scalability of IoT networks, allowing for the easy addition of new devices without significant changes to the existing infrastructure. BLE also supports mesh networking, introduced with Bluetooth 5.0, which enhances the range and reliability of IoT networks by allowing devices to communicate directly with each other rather than relying solely on a central hub. The robust security features of BLE, including AES-128 encryption, ensure that data transmitted between devices are secure. This is particularly important in IoT applications that handle sensitive information, such as personal health data or home security systems. The secure communication provided by BLE helps to protect against unauthorized access and cyber threats, ensuring the integrity and confidentiality of the data. Moreover, the support of BLE for various data transfer modes and customization options allows it to meet the specific needs of different IoT applications. For instance, the advertising and broadcasting capabilities of BLE enable devices to share data with multiple receivers without establishing a dedicated connection, which is useful for applications like location-based services and asset tracking. The widespread adoption of BLE in consumer electronics also contributes to its integration into the IoT framework. Many smartphones, tablets, and computers are equipped with BLE, allowing for easy interaction and control of IoT devices through commonly used interfaces. This ubiquity enhances user convenience and promotes the adoption of IoT solutions. Bluetooth Low Energy 6.0 introduces features (see Figure 3) that are expected to address critical IoT challenges, while potentially opening new possibilities for advanced applications. The introduction of “Bluetooth Channel Sounding” could revolutionize IoT asset tracking and security applications through its distance awareness capabilities, which may prove particularly valuable in industrial IoT environments where asset location accuracy is crucial. The “Decision-Based Advertising Filtering” feature is anticipated to enhance power management in IoT deployments by potentially reducing unnecessary scanning operations, which could be especially beneficial in large-scale sensor networks where battery life is paramount. This improvement may help address one of the primary challenges in IoT implementations—power consumption. The “Monitoring Advertisers” capability is expected to bring benefits to IoT device management by potentially enabling more efficient network maintenance and reducing connection overhead, which could prove valuable in smart building and industrial automation scenarios. Enhanced audio capabilities with improved LC3 codec support might enable new IoT use cases in voice-controlled systems and industrial communication devices, while potentially maintaining power efficiency. The negotiable “Frame Space Update” feature could provide new flexibility in managing transmission intervals, potentially allowing IoT devices to optimize their communication patterns based on specific application requirements. The “LL Extended Feature Set” is designed to facilitate better device interoperability, which may help address challenges in heterogeneous IoT environments. Additionally, the improved Isochronous Adaptation Layer’s latency capabilities could benefit time-critical IoT applications in industrial automation and healthcare monitoring. These advancements may collectively position BLE 6.0 as a more robust and versatile protocol for IoT applications, potentially offering improved efficiency, reliability, and functionality while maintaining the low-power characteristics essential for IoT deployments. In summary, with BLE, its low power consumption, short-range communication capabilities, simple protocol stack, robust security features, and support for mesh networking make it an ideal technology for integrating into the IoT framework. By providing reliable, efficient, and secure connectivity, BLE enhances the functionality and scalability of IoT systems, enabling a wide range of innovative applications that improve the efficiency, convenience, and quality of life for users.

### 4.3. Use Cases: Specific Applications and Case Studies of BLE in IoT

Bluetooth Low Energy has been widely adopted across various industries, demonstrating its versatility and efficiency in numerous IoT applications. Here are some specific use cases and case studies that highlight the impact of BLE in different domains:

#### 4.3.1. Smart Homes

In smart home environments, BLE is extensively used to connect and control a variety of devices, enhancing convenience and automation [41]. For instance, smart lighting systems use BLE to allow users to control lights via smartphone apps [42] or voice commands. Smart thermostats, such as the Nest Thermostat, utilize BLE to communicate with sensors placed throughout the home to maintain optimal temperatures efficiently. According to the study by Stopps and Touchie in [43], the use of smart thermostats enhances energy savings when compared to operating without a schedule. BLE-enabled smart locks provide secure, keyless entry to homes, enhancing security and ease of access. Bapat et al. [44] in their study propose a possible solution for designing smart-locks with an increased level of security. They propose the combination of Image Steganography and Cryptography in order to overcome the vulnerabilities of the BLE protocol. These devices create an interconnected ecosystem that improves energy efficiency and user comfort. The authors in this study [45] implemented a Bluetooth Mesh Network to enhance smart home functionality, emphasizing security and efficient data transmission. The system employed various types of nodes, including remote nodes, relay nodes, provisioner nodes, and lock nodes, using the nRF52840 Bluetooth System-on-Chip (SoC) module. A mesh topology facilitates many-to-many communication, enabling the real-time control of smart locks and other devices through a cloud-connected gateway. Experimental setups tested transmission across single rooms, multiple rooms, and even floors, achieving reliable data transfer of up to 28.7 m with minimal packet loss in ideal conditions. Power-saving algorithms reduced relay node energy consumption by approximately 36%, optimizing system efficiency. This case study highlights BLE mesh networks’ scalability, cost effectiveness, and low energy consumption, making them ideal for smart home applications where seamless integration of IoT devices is essential.

#### 4.3.2. Automotive

In the automotive sector, BLE technology has been increasingly adopted to enhance convenience, safety, and overall driving experience. One of the primary applications of BLE in automobiles is smart vehicle access, where a driver’s smartphone acts as a virtual key, enabling secure functions such as locking, unlocking, and starting the engine through bidirectional communication between the phone and the vehicle. This technology not only improves convenience for users but also integrates personalized entertainment and connected experiences into the driving environment [46]. Another critical application is wireless sensor connectivity, which allows for the replacement of traditional wired connections with BLE-enabled sensors. This shift reduces vehicle weight, enhances design flexibility, and can lead to improved fuel efficiency by minimizing wiring complexity [46]. Additionally, BLE supports Tire Pressure Monitoring Systems (TPMSs) [47] and Wireless Battery Management Systems (WBMSs) [48], providing real-time data on tire conditions and battery health directly to smartphones of the drivers. This capability not only enhances safety but also facilitates proactive maintenance by alerting users to potential issues before they escalate. Another notable use case is the integration of BLE-based wireless charging systems in modern vehicles [49,50]. This enables seamless phone charging while maintaining vehicle-specific safety features such as hands-free operation, voice commands, and emergency alerts. Additionally, BLE connectivity allows for the remote monitoring and control of automotive systems, including engine performance monitoring, fuel efficiency tracking, and real-time traffic updates. Furthermore, BLE-enabled Car-to-Car (C2C) or Car-to-Infrastructure (C2I) communication can be leveraged to enable Advanced Driver Assistance Systems (ADASs), improved road safety, and enhanced parking experiences. The use of BLE in the automotive industry not only simplifies vehicle management but also opens up new possibilities for innovative services and applications that improve daily driving routines. As vehicles become increasingly sophisticated, the integration of BLE technology is vital for developing smart features that prioritize user experience and operational efficiency.

#### 4.3.3. Healthcare

BLE plays a crucial role in healthcare by enabling continuous monitoring and data collection from medical devices and wearables. For example, wearable fitness trackers like Fitbit and Garmin use BLE to sync health data, such as heart rate and activity levels, to smartphones and health apps [51]. In clinical settings, BLE is extensively used in medical devices like glucose monitors and pulse oximeters to transmit patient data to healthcare providers in real-time as highlighted in studies on telemedicine platforms integrating BLE for remote chronic disease management [52]. This technology underpins Remote Health Monitoring Systems (RHMSs) that allow patients, especially the elderly or those with chronic conditions, to be monitored at home, reducing hospital visits and enhancing disease management outcomes [53]. Such systems employ BLE-equipped sensors, including ECG devices and pulse oximeters, to capture and transmit critical physiological data, enabling real-time interventions. Case studies have shown that BLE-enabled devices significantly enhance remote patient monitoring, reducing hospital readmissions and improving chronic disease management by ensuring consistent communication between patients and care teams [51,52,53]. Another case study [54] conducted in New Zealand examined the use of BLE technology to enhance contact tracing efforts during the COVID-19 pandemic. A 7-day trial involved BLE-enabled cards, worn by participants in a small community, to automatically detect and record proximity events. The study compared this technology to traditional contact tracing methods, which rely on self-reported interactions. The BLE cards demonstrated higher consistency in detecting interactions, capturing 88% of contact events between paired devices, compared to 64% through self-reports. However, the BLE system also faced challenges such as participant non-compliance and false positives. The findings highlighted the potential of BLE as a supplemental tool to traditional methods, enhancing the speed and accuracy of contact tracing while underscoring the need for measures to ensure better adoption and reliability.

#### 4.3.4. Wearables

The wearable technology market heavily relies on BLE due to its low power consumption and reliable connectivity. Smartwatches, such as the Apple, Android and Garmin watches, use BLE to communicate with smartphones, providing notifications, health tracking, and other functionalities without draining battery life rapidly. BLE is also used in specialized wearables, such as hearing aids [55], which benefit from its low power profile to extend battery life while maintaining high-quality audio streaming. The use of BLE in wearables has expanded beyond consumer applications to include industrial wearables that monitor worker health and safety, demonstrating the broad applicability of BLE [56].

#### 4.3.5. Retail and Proximity Marketing

BLE beacons are widely used in retail environments to enhance the shopping experience through proximity marketing [57,58,59]. Retailers deploy BLE beacons throughout stores to send personalized offers and product information to customers’ smartphones as they move through different sections. For example, a customer passing by the electronics section might receive a notification about a discount on headphones. Case studies have shown that this targeted marketing approach increases customer engagement and sales [60,61]. BLE beacons also help in indoor navigation, guiding customers to specific products or sections within large stores. The authors in case study [62] proposed a BLE location-based indoor positioning system for real-time asset tracking in warehouse environments. The system architecture comprised BLE beacons attached to assets, a Raspberry Pi 3 model B as the Bluetooth signal receiver, and a central data processing unit. Beacons broadcast signals at regular intervals, with receivers gathering these signals to estimate asset locations using trilateration. Advanced signal filtering techniques, including a Kalman-LULU filter, were employed to reduce noise and improve localization accuracy. Experiments demonstrated the system’s effective range at 9.5 m with a transmission power of 0 dBm, achieving a root mean square error of 22.42% a significant improvement over traditional methods. Furthermore, the study addressed challenges such as interference from metal shelving, recommending beacon placement far from metallic structures to mitigate signal attenuation. This innovative approach highlighted the cost effectiveness and scalability of BLE for industrial asset management applications.

#### 4.3.6. Industrial Automation

BLE has become a pivotal technology in the realm of industrial automation, particularly within the IoT ecosystem. Its design prioritizes low power consumption while maintaining robust communication capabilities, making it suitable for time-sensitive applications in industrial settings. For instance, research indicates that with BLE, its ultra-low energy properties and compatibility with various mobile devices enable efficient data transmission in environments that demand real-time responsiveness, such as industrial process automation [63]. A study highlights the potential of BLE to meet the stringent requirements of Industrial Wireless Sensor Networks (IWSNs) and Industrial Internet of Things (IIoT), emphasizing its superior throughput performance compared to other low-energy wireless protocols like ZigBee [63]. Furthermore, the flexible network topologies and built-in security mechanisms of BLE enhance its applicability across diverse industrial scenarios, from smart manufacturing systems to remote monitoring solutions [64,65]. As industries increasingly adopt BLE for automation purposes, ongoing research is crucial to address challenges related to reliability, latency, and energy efficiency in these critical applications [66].

#### 4.3.7. Smart Cities

BLE technology plays a pivotal role in the development of smart cities by enabling a multitude of applications that enhance urban living. This technology is also a key enabler in the development of smart city infrastructure. BLE beacons and sensors are deployed in urban areas to monitor environmental conditions, such as air quality and noise levels, providing real-time data to city management systems. This information helps in making informed decisions to improve urban living conditions. BLE beacons, for instance, are utilized for location-based services, indoor navigation, and proximity marketing, allowing cities to create more interactive environments for residents and visitors alike. These beacons operate by broadcasting signals that can be detected by smartphones and other BLE-enabled devices, facilitating real-time information exchange and engagement with the urban infrastructure [67,68]. Additionally, BLE is used in smart parking systems, where sensors detect available parking spots and relay this information to drivers via mobile apps, reducing traffic congestion and emissions. Case studies from cities that have implemented BLE-based smart parking solutions report increased efficiency and convenience for residents. Moreover, the low power consumption and cost effectiveness of BLE make it an ideal choice for deploying a vast network of sensors and devices across urban areas, which can monitor environmental conditions, manage traffic flow, and optimize energy usage in smart lighting systems [68,69]. As cities increasingly adopt IoT technologies, the integration of BLE not only enhances operational efficiency but also addresses the challenges related to security and privacy, ensuring that smart city initiatives are both innovative and sustainable [70]. Recent studies highlight the importance of machine learning in conjunction with Bluetooth and other short-range wireless technologies for optimizing smart transportation systems within smart cities, showcasing the synergy between these technologies in enhancing urban mobility and infrastructure management [71].

#### 4.3.8. Summarize

These use cases and case studies illustrate the diverse applications of BLE in the IoT ecosystem. The low power consumption, reliable connectivity, and versatility of BLE make it an essential technology for creating interconnected, intelligent systems across various industries, improving efficiency, convenience, and overall quality of life.

### 4.4. BLE and the Sustainable Development Goals of United Nations

The growing emphasis on sustainability in technology development has brought attention to how low-power wireless solutions can contribute to the United Nations’ Sustainable Development Goals (SDGs) [72]. BLE, with its inherent focus on energy efficiency and interoperability, aligns strongly with several SDGs, notably SDG 7, SDG 11, SDG 12, and SDG 13. In the following subsections, we outline how BLE technology contributes to these global objectives.

#### 4.4.1. SDG 7: Affordable and Clean Energy

The integration of BLE with solar-powered systems demonstrates a significant step toward achieving SDG 7, which emphasizes access to affordable, reliable, and sustainable energy. For example, solar-powered agricultural robots equipped with BLE for remote control and monitoring have been shown to optimize energy use by harnessing renewable energy sources for operations such as seed sowing, pesticide spraying, and grass cutting. These systems, as illustrated by the work on autonomous agricultural robots, rely on solar panels to charge batteries that drive the robot’s mechanisms while minimizing the environmental footprint [73]. Similarly, BLE-enabled smart home automation systems powered by solar energy provide an eco-friendly approach to energy management, allowing remote monitoring and efficient energy utilization through IoT integration. Such systems reduce reliance on non-renewable energy sources while promoting sustainable energy practices, making them a valuable tool in advancing the SDG 7 goals [74].

#### 4.4.2. SDG 11: Sustainable Cities and Communities

Bluetooth technology, as part of IoT systems, significantly supports the SDG 11 goal, by enabling smart, sustainable urban solutions. The authors in [75] highlight the role of IoT in smart cities through applications like environmental monitoring, smart transportation, and waste management. Bluetooth-enabled IoT systems facilitate real-time data collection and communication, such as integrating mobile sensors to monitor air quality and optimize traffic flows. Key findings indicate that Bluetooth technology enhances energy efficiency, reduces urban environmental impacts, and supports the creation of adaptive, resilient urban infrastructures. These advancements illustrate how Bluetooth contributes to sustainable urban development, aligning with the SDG 11 goals

#### 4.4.3. SDG 12: Responsible Consumption and Production and SDG 13: Climate Action

The integration of Bluetooth technology into Green IoT (G-IoT) systems directly supports SDG 12 and SDG 13 by promoting sustainable consumption, reducing waste, and mitigating environmental impact. The Green IoT Bluetooth Hotmeal Container (GIoT-BHMC) [76] exemplifies these contributions by utilizing rechargeable batteries and low-power Bluetooth components to reduce energy consumption and e-waste. This approach aligns with the SDG 12’s focus on responsible resource use while fostering eco-friendly practices. Furthermore, the energy-efficient design of GIoT systems helps lower carbon emissions and minimizes environmental footprints, advancing SDG 13’s climate action goals. By embedding sustainability into IoT technologies, these innovations demonstrate how smart solutions can create greener, more sustainable communities.

#### 4.4.4. Future Perspectives for BLE and Sustainability

As BLE continues to evolve (see Section 3.3), further improvements in power efficiency, data rate, and security will broaden its applicability in sustainable technology solutions. Future BLE-based systems could incorporate advanced machine learning on edge devices for proactive resource management, thereby extending battery life and minimizing energy consumption even further. Incorporating these SDG-focused perspectives into ongoing research and development ensures that BLE is not only advancing technologically but also driving responsible and sustainable innovation across diverse IoT contexts.

## 5. Technical Challenges and Solutions

### 5.1. Challenges: Common Technical Challenges in Implementing BLE in IoT

Implementing Bluetooth Low Energy in IoT applications presents several technical challenges that need to be addressed to ensure efficient and reliable performance.

#### 5.1.1. Power Management

One of the significant technical challenges in implementing BLE in IoT devices is power management [77]. BLE devices are designed to be low power, but they still require a reliable power source to maintain connectivity and perform tasks. The limited battery life of IoT devices can lead to frequent replacements or recharging, which can be inconvenient and costly. Moreover, the power consumption of BLE devices can vary significantly depending on factors such as transmission power, data rate, and duty cycle, making it challenging to optimize power management. Additionally, the use of coin cell batteries in many IoT devices further exacerbates the power management challenge due to their limited capacity and voltage constraints. As a result, designers and developers must carefully balance power consumption with performance requirements to ensure the reliable and efficient operation of BLE-based IoT devices.

#### 5.1.2. Interference and Coexistence

BLE operates in the 2.4 GHz ISM band, which is shared by many other wireless technologies such as Wi-Fi, Zigbee, and microwave ovens [78,79]. This can lead to interference, causing signal degradation, packet loss, and reduced communication range. Moreover, the increasing density of IoT devices in a given area can exacerbate the problem, making it challenging to ensure reliable and efficient communication between BLE devices. The coexistence of BLE with other wireless technologies is further complicated by the varying transmission power, data rate, and duty cycle of these technologies, which can create complex interference scenarios that are difficult to predict and mitigate. As BLE technology is increasingly adopted in critical applications, such as healthcare devices that require reliable communication, ensuring robust coexistence with other technologies becomes essential to maintain low latency and uninterrupted service [78,79]. Ensuring that BLE devices can coexist with other wireless technologies without significant performance degradation is a key challenge.

#### 5.1.3. Scalability

One of the significant challenges in implementing BLE in Internet of Things applications is scalability [80,81,82]. As the number of devices increases, the complexity of the network also increases, leading to problems with connectivity, data transmission, and power consumption. In a dense IoT environment, multiple devices competing for the same frequency band can cause interference, packet loss, and decreased throughput, ultimately affecting the overall performance of the system. In addition, as the network expands, the need for more gateways, routers, or hubs to manage device connections arises, which can lead to increased infrastructure costs, power consumption, and maintenance requirements. The challenge of scalability is further exacerbated by the limited capacity of BLE devices to handle a large number of connections, making it essential to develop strategies to efficiently manage device interactions and optimize network performance.

#### 5.1.4. Security

The implementation of Bluetooth Low Energy in IoT devices also raises significant security concerns [83,84]. One of the major challenges is the risk of unauthorized access to sensitive data transmitted over BLE connections. Since BLE devices often operate in a low-power, low-data-rate mode, they may not have the resources to implement robust security protocols, making them vulnerable to attacks [85]. A wide variety of attacks against BLE have been studied, such as eavesdropping, Man-in-the-Middle (MitM), MAC address spoofing, cryptographic vulnerabilities, Denial of Service (DoS), distortion, and surveillance. Moreover, device fingerprinting attacks, exploiting static MAC addresses and UUIDs, have enabled tracking individuals even when anti-tracking measures like MAC randomization are in place [83]. Additionally, the use of BLE in IoT devices can also introduce new attack vectors, such as bluejacking and bluesnarfing, which can allow attackers to steal sensitive information or take control of devices [86]. Ensuring secure pairing and data encryption without compromising performance or user experience is a complex task. Furthermore, the increasing number of connected devices in the IoT ecosystem also increases the potential for large-scale attacks, making it essential to address these security challenges to ensure the integrity and confidentiality of data transmitted over BLE connections.

#### 5.1.5. Data Throughput and Latency

Data throughput and latency are critical factors that significantly influence the performance and user experience of BLE in IoT applications. The BLE design prioritizes low power consumption over data transfer speed and latency. This trade-off becomes a challenge in scenarios requiring real-time data communication or handling large volumes of data, such as in industrial IoT or multimedia applications. Moreover, interference from other wireless technologies operating in the crowded 2.4 GHz ISM band can exacerbate latency and reduce throughput. The limitations in the BLE payload size, connection interval settings, and the impact of packet loss due to environmental factors or device mobility further complicate reliable communication. BLE 5 and 6 have been designed to enhance BLE 4 by incorporating additional features that cater to high data rate IoT applications. These upgraded versions offer various Bluetooth Physical Layer (PHY) configurations, including 1M PHY, 2M PHY, and Coded PHY, each with its own balance between data throughput and range. Notably, the LE 2M feature stands out for enabling the highest achievable data rate, reaching approximately 1.3 Mbps in practical use [87]. Additionally, the discovery process in BLE networks can introduce delays; improper parameter settings during device discovery can exacerbate latency issues, resulting in longer wait times for devices to connect and communicate [88]. These challenges are particularly pronounced in environments with multiple devices competing for bandwidth, leading to increased contention and further delays in data transmission. Optimizing BLE to meet the specific data requirements of various applications while maintaining low power consumption is essential.

#### 5.1.6. Interoperability

Interoperability is a critical challenge in implementing BLE technology within the IoT ecosystem. With the proliferation of diverse devices from multiple manufacturers, ensuring seamless communication among them has become increasingly complex. Variability in BLE stack implementations, differences in firmware, and non-uniform adherence to Bluetooth standards can lead to inconsistencies and device incompatibility. Additionally, IoT environments often integrate devices with varying resource constraints and communication protocols, further complicating the establishment of a standardized framework. The lack of robust testing and certification processes for ensuring interoperability exacerbates the issue, leading to fragmented user experiences and limiting the potential of BLE in realizing a truly interconnected IoT network [89].

#### 5.1.7. Range Limitations

BLE is widely adopted in the IoT ecosystem due to its low power consumption and cost effectiveness. While BLE offers a reasonable range for many applications, extending the range without significantly increasing power consumption remains a challenge. Typically, BLE devices have an operational range between 10 and 50 m, which can be insufficient for applications like smart cities or large industrial environments [23]. Factors such as physical obstructions, environmental interference, and regulatory constraints further exacerbate these range limitations, potentially leading to unreliable communication and reduced network performance. Applications requiring extensive coverage need solutions to overcome the range limitations of BLE.

#### 5.1.8. Complexity of Mesh Networking

The implementation of BLE mesh networking in IoT ecosystems poses significant technical challenges [3]. One of the primary concerns is the complexity of mesh networking itself, which involves multiple devices communicating with each other in a web-like topology. As the number of devices increases, the network becomes increasingly complicated, leading to issues such as packet loss, latency, and reduced network reliability. Furthermore, the synchronization of data transmission and reception among numerous devices can be difficult to achieve, resulting in decreased overall network performance. The complexity of mesh networking also introduces security risks, as a single vulnerable device can potentially compromise the entire network. Additionally, the lack of standardization in mesh networking protocols can lead to interoperability issues between devices from different manufacturers, hindering the widespread adoption of BLE-based IoT solutions. Managing the network topology, ensuring the efficient routing of messages, and maintaining network stability and performance are challenging tasks that require sophisticated algorithms and robust network management [90].

#### 5.1.9. Firmware Updates and Maintenance

Implementing firmware updates and maintenance for a large number of BLE-enabled devices within the IoT ecosystem presents several technical challenges. Firstly, the constrained memory and processing capabilities of many IoT devices can impede the storage and execution of firmware updates, particularly when updates are substantial or require complex installation procedures. Secondly, maintaining a stable and secure connection during the update process is challenging, especially in environments with intermittent connectivity, which can lead to incomplete or corrupted firmware installations [91]. Additionally, ensuring the security of firmware updates is critical; unauthorized access or tampering during the update process can introduce vulnerabilities, compromising the entire IoT network [92]. These challenges necessitate robust solutions to ensure efficient and secure firmware management in BLE-enabled IoT devices.

#### 5.1.10. Summarize

Addressing these challenges is essential for the successful implementation and operation of BLE in IoT applications. Solutions to these issues involve continuous advancements in technology, robust design practices, and adherence to standards and best practices in IoT development.

### 5.2. Solutions: Proposed and Existing Solutions to These Challenges

To address the various challenges in implementing Bluetooth Low Energy in IoT applications, several solutions have been proposed and developed, focusing on enhancing power management, interference mitigation, scalability, security, data throughput, interoperability, range, mesh networking, and firmware updates.

#### 5.2.1. Power Management Solutions

To address the power management challenges in BLE-based IoT devices, several solutions have been proposed and implemented [77,93]. One approach is to use advanced power management techniques such as duty cycling, where the device is switched on and off periodically to reduce power consumption [90,94]. Another solution is to leverage low-power modes, such as sleep or hibernate modes, which can significantly reduce power consumption when the device is not transmitting or receiving data. Additionally, the use of energy harvesting technologies [93], such as solar or vibration-based energy harvesting, can help supplement the battery life of IoT devices. Furthermore, advances in semiconductor technology have led to the development of low-power System on Chip (SoC) solutions that integrate BLE radios with microcontrollers and other peripherals, reducing power consumption and improving overall efficiency. By adopting these solutions, designers and developers can create BLE-based IoT devices that are not only energy efficient but also reliable and cost effective, enabling widespread adoption in various IoT applications.

#### 5.2.2. Interference and Coexistence Mitigation

To address the challenges of interference and coexistence in BLE implementations, several strategies have been proposed and developed [95,96]. One effective approach is the use of the Frequency Hopping Spread Spectrum (FHSS), which allows BLE devices to rapidly switch between different channels within the 2.4 GHz band. This technique helps mitigate the effects of interference by enabling devices to “hop” to clearer channels when disturbances are detected [78,95]. Furthermore, advancements in Adaptive Frequency Hopping (AFH) techniques enhance this process by continuously monitoring channel conditions and dynamically selecting optimal channels based on real-time interference levels [97]. Continuous channel monitoring is crucial for maintaining robust connections in unpredictable environments [95]. Additionally, manufacturers are encouraged to adhere strictly to Bluetooth standards while incorporating high-quality components that improve resilience against interference. Future developments may also explore higher-frequency bands that offer more channels and reduced interference potential, thereby enhancing the overall reliability of BLE in the IoT ecosystem [78,79].

#### 5.2.3. Scalability Solutions

To address the scalability challenges in BLE-based IoT systems, several solutions have been proposed. One approach is to implement mesh networking topologies, which enable multiple devices to act as relays, allowing data to be transmitted over longer distances and reducing the need for a central hub or gateway [81]. Another solution is to utilize techniques such as frequency hopping and adaptive frequency allocation to minimize interference and optimize channel utilization. Additionally, advancements in BLE 5.0 and later versions have introduced features like increased throughput, improved coexistence mechanisms, and enhanced connection capacities, enabling more efficient device interactions and better support for large-scale IoT deployments [80,97]. Furthermore, the use of cloud-based services and fog computing can help offload processing tasks from edge devices, reducing the computational burden and enabling more scalable and efficient management of IoT networks. By adopting these solutions, developers can design and deploy scalable BLE-based IoT systems that can efficiently handle a growing number of devices and applications.

#### 5.2.4. Security Enhancements

To address the security challenges associated with implementing BLE in IoT devices, several solutions have been proposed and implemented. One approach is to use secure pairing mechanisms, such as Elliptic Curve Diffie–Hellman (ECDH) key exchange, to establish secure connections between devices [98]. Enhancing the pairing process is critical; using more secure methods such as OOB pairing can significantly reduce the risk of MitM attacks by ensuring that both devices authenticate each other through a separate channel. Implementing strong encryption protocols, such as AES-128, ensures that data transmitted over BLE connections remain confidential and secure from eavesdropping [99]. Additionally, adopting Pre-Shared Keys (PSKs) during manufacturing can facilitate secure automatic pairing without user intervention, thus enhancing security in environments where user interaction is impractical. Another solution is to incorporate Public Key Infrastructure (PKI)-based certificate management for device authentication and authorization. Rather than relying solely on PKI in general, this approach utilizes digital certificates, such as X.509, to validate a device’s identity, leveraging a trusted Certification Authority (CA). During the device provisioning stage, the IoT infrastructure issues unique certificates that bind a public key to each device. When devices attempt to access sensitive data or network resources, their certificates are verified against the CA, and only authorized devices are granted access. This layered approach couples PKI with an authorization protocol or access control mechanism, ensuring that all communication remains restricted to verified participants. Furthermore, firmware updates and patches can be used to fix vulnerabilities and prevent attacks. The use of secure coding practices, such as secure boot mechanisms and secure firmware storage, can also help prevent attacks on BLE-enabled IoT devices. Moreover, utilizing sophisticated threat modeling methods such as STRIDE [100], or deploying other advanced Intrusion Detection and Prevention Systems (IDPS), can significantly enhance the detection and prevention of large-scale attacks within the IoT ecosystem, thereby safeguarding the security and integrity of data transmitted via BLE connections.

#### 5.2.5. Data Throughput and Latency Optimization

Optimizing data throughput and latency in BLE-based IoT systems requires a multifaceted approach. Enhancements that take place in the latest Bluetooth versions, featuring increased Maximum Transmission Unit (MTU) sizes, faster data transfer rates, and improved channel selection algorithms, address many throughput and latency issues. Adaptive connection parameters, like dynamically adjusting the connection interval and slave latency based on application demands, can balance energy efficiency with performance. Leveraging advanced error correction techniques and employing channel hopping to mitigate interference further enhances data reliability. Additionally, application-layer optimizations, such as aggregating data or compressing payloads, can maximize throughput without requiring significant changes to the BLE protocol stack. These combined strategies enable BLE to meet the diverse demands of modern IoT ecosystems.

#### 5.2.6. Interoperability Solutions

Addressing interoperability challenges in BLE-based IoT systems requires adherence to standards, rigorous testing, and the development of universal frameworks. Adopting Bluetooth SIG’s interoperability guidelines and ensuring consistent firmware updates across devices can mitigate compatibility issues. Advanced testing platforms, such as interoperability test beds and automated tools, enable manufacturers to evaluate device compatibility under real-world conditions. Emerging solutions, like middleware layers and unified communication protocols, bridge the gap between heterogeneous devices, facilitating seamless interaction. Collaborative industry efforts to establish open-source BLE stacks and shared development platforms can accelerate innovation while ensuring a standardized, interoperable IoT ecosystem [101].

#### 5.2.7. Range Extension Techniques

To address the above-mentioned range limitations, several techniques have been proposed and implemented. One viable approach to expanding coverage areas is by implementing relay nodes or repeaters. Another effective method is utilizing the long-range feature introduced in Bluetooth 5, known as Coded PHY, which enhances range by adding redundancy to the transmitted data, thereby improving the receiver’s ability to decode signals over longer distances [22]. Additionally, increasing the transmit power can extend the range; however, this approach must be balanced against battery life considerations and regional regulatory limits. Implementing mesh networking allows multiple BLE devices to relay data, effectively expanding the coverage area without significantly increasing power consumption [3,90]. Hybrid solutions combining BLE with other wireless technologies, such as LoRa or cellular networks, can also provide extended range capabilities. These solutions enable BLE to meet the diverse range requirements of various IoT applications.

#### 5.2.8. Mesh Networking Solutions

To address the challenges associated with BLE mesh networking, several solutions have been proposed and implemented. One approach is the use of standardized mesh networking protocols, such as the Bluetooth Mesh Profile, which enables seamless communication between devices from different vendors [102]. Another solution involves the implementation of advanced routing algorithms, such as flooding and mesh routing, to optimize data transmission and reduce latency [103]. Advanced routing protocols and self-healing mechanisms ensure continuous network operation even in the event of node failures. Additionally, the adoption of techniques like network partitioning and device grouping can help simplify the mesh topology, reducing the complexity and improving the overall performance of the network. Furthermore, the use of security protocols like encryption and secure key exchange can mitigate the risks associated with mesh networking, ensuring the integrity and confidentiality of data transmission [104]. Finally, the development of software tools and frameworks that simplify the design, deployment, and management of BLE mesh networks can facilitate the adoption of this technology in IoT ecosystems, enabling the creation of more efficient, scalable, and reliable connected systems.

#### 5.2.9. Firmware Update and Maintenance Solutions

To address the above-mentioned challenges, several solutions have been proposed and implemented. Over-the-Air (OTA) firmware updates have emerged as a practical approach, allowing remote and wireless distribution of firmware, thereby eliminating the need for physical access to devices. Implementing delta updates, which involve transmitting only the differences between the current and new firmware versions, can mitigate memory constraints by reducing the size of the data transfer and minimizes disruption [91]. To enhance security, employing encryption and authentication mechanisms during the update process ensures that only authorized firmware is installed, protecting against potential threats. Additionally, ensuring robust update verification and rollback capabilities protects devices from faulty updates and maintains system integrity [105]. These strategies collectively contribute to more efficient and secure firmware update processes in BLE-enabled IoT devices.

#### 5.2.10. Summarize

Addressing these challenges is essential for the successful implementation and operation of BLE in IoT applications. Solutions to these issues involve continuous advancements in technology, robust design practices, and adherence to standards and best practices in the development of IoT. The interplay between different BLE characteristics and solutions creates complex trade-offs and synergies that significantly impact implementation decisions. For example, while increasing transmission power can improve range and reduce latency, it directly conflicts with power management goals and device longevity. Advanced security implementations such as PKI-based authentication and encryption enhance data protection but introduce additional processing overhead, memory requirements, and latency. Mesh networking demonstrates positive synergies by simultaneously addressing range limitations, scalability challenges, and network resilience, although at the cost of increased network complexity and power consumption at relay nodes. The adoption of adaptive frequency hopping creates multiple benefits. It mitigates interference, improves power efficiency by reducing retransmissions, and enhances data throughput, exemplifying a solution with positive cascading effects. However, implementing sophisticated firmware update mechanisms with delta updates and security features addresses maintenance challenges while potentially straining device memory and processing capabilities. The use of duty cycling for power management can extend battery life but may impact real-time performance and increase latency. Interoperability solutions through standardization and unified protocols can simplify device integration but may limit innovation and specialized optimizations. Modern BLE implementations must carefully balance these interconnected trade-offs while leveraging potential synergies, particularly considering that improvements in one area often create challenges in others. The key to successful implementation lies in understanding these relationships and optimizing the balance based on specific application requirements, environmental constraints, and use case priorities.

## 6. Future Directions and Trends

### 6.1. Emerging Trends: Upcoming Advancements in BLE Technology and Its Future Role in IoT

Bluetooth Low Energy technology continues to evolve, driven by the growing demands of the IoT ecosystem. Emerging trends and upcoming advancements in BLE technology promise to enhance its capabilities, making it even more integral to the future of IoT.

#### 6.1.1. Increased Data Rates and Efficiency

Future versions of BLE are expected to offer higher data rates while maintaining low power consumption. The adoption of features like LE Audio’s Isochronous Channels and improvements in modulation techniques contribute to more efficient data transmission and reduced latency, enabling seamless real-time communication. Furthermore, these advancements pave the way for high-density IoT ecosystems, where multiple devices can coexist and exchange information reliably without compromising performance. As the demand for high-bandwidth applications such as video streaming, AR, and advanced sensor networks grows, the ability of BLE to balance speed, efficiency, and energy savings will solidify its position as a cornerstone technology in the IoT landscape.

#### 6.1.2. Enhanced Security Features

As IoT devices become more ubiquitous, the importance of robust security measures grows. Upcoming BLE advancements will focus on enhancing security features to protect against increasingly sophisticated cyber threats. This includes improved encryption algorithms, advanced authentication methods, and secure boot processes. The integration of quantum-resistant cryptographic techniques is also being explored to future-proof BLE devices against potential quantum computing threats.

#### 6.1.3. Better Location Services

The role of BLE in location-based services is set to expand with more precise and reliable positioning capabilities. Enhancements in direction finding and Angle-of-Arrival (AoA) technologies will allow for centimeter-level accuracy in indoor positioning systems. This will enable a wide range of applications, from asset tracking in warehouses to enhanced navigation in smart buildings and retail environments.

#### 6.1.4. Improved Power Management

Future BLE developments will continue to focus on reducing power consumption and extending battery life. Innovations in energy harvesting, such as solar and kinetic energy solutions, will complement the low power profile of BLE, enabling devices to operate indefinitely without battery replacements. Additionally, more efficient power management algorithms will dynamically adjust power usage based on the device’s operating conditions and communication requirements.

#### 6.1.5. Mesh Networking Enhancements

Advancements in BLE mesh networking will improve the scalability, reliability, and efficiency of IoT networks. Future BLE mesh protocols will offer better support for large-scale networks with thousands of nodes, ensuring seamless communication and data transfer across extensive areas. Self-healing and adaptive routing algorithms will enhance network robustness, allowing the network to automatically adjust to changes and failures without manual intervention.

#### 6.1.6. Integration with Other Technologies

BLE will increasingly be integrated with other wireless technologies to create hybrid solutions that leverage the strengths of multiple communication standards. For example, combining BLE with Wi-Fi, cellular, or LoRa technologies will enable comprehensive IoT solutions that offer both short-range and long-range communication capabilities. This integration will support more complex and diverse IoT ecosystems, such as smart cities and industrial IoT applications.

#### 6.1.7. Advanced Audio Capabilities

The release of Bluetooth Low Energy 6.0 in August 2024 has introduced groundbreaking advancements in audio capabilities, solidifying its role in the evolving IoT ecosystem. BLE 6.0 builds on the LC3 codec from Bluetooth 5.2, delivering unprecedented audio quality with ultra-low latency while maintaining energy efficiency. Notable features include enhanced multi-stream audio support, allowing seamless synchronization across multiple devices, and superior broadcast capabilities for sharing high-fidelity audio streams with numerous receivers [106]. These advancements cater to diverse applications such as augmented reality (AR) and Virtual Reality (VR) environments, smart hearing aids, and wireless earbuds, offering an immersive and responsive audio experience. Furthermore, BLE 6.0 paves the way for innovative use cases in voice-assisted IoT devices and smart home systems, ensuring robust and real-time audio communication that elevates user interaction in the interconnected world.

#### 6.1.8. AI and ML Integration

The integration of artificial intelligence (AI) and machine learning (ML) with BLE is poised to revolutionize the IoT ecosystem. While AI broadly refers to intelligent systems capable of reasoning, problem-solving, and autonomous decision-making, ML is a subset of AI that specifically involves training models to learn from data and make predictions or decisions without explicit programming. ML is proving to be a powerful tool for enhancing the capabilities of BLE technology, particularly in the areas of indoor positioning, distance estimation, and security. The paper [107] shows how ML models, including linear regression, decision trees, random forests, and neural networks, can significantly reduce distance estimation errors and improve proximity classification accuracy when trained on the BLE Received Signal Strength Indicator (RSSI) and transmission power data. Another study [108] demonstrates the effectiveness of ML in building highly accurate indoor positioning systems that leverage RSSI measurements from strategically placed BLE beacons. This approach can achieve sub-meter accuracy, addressing the limitations of GPS in indoor environments. Furthermore, the authors in [109] highlight the crucial role of ML in detecting and mitigating security threats in BLE networks. By analyzing BLE packet features and applying reconstruction and classification techniques, researchers can successfully identify and classify malicious packets, protecting BLE devices from MitM attacks. AI techniques offer a promising solution for tackling the real-world complexities of BLE signal propagation in indoor environments. For instance, the research presented in [110] introduces an AI-powered approach to detect and compensate for signal attenuation caused by human obstacles during BLE trilateration. By training Artificial Neural Networks (ANNs) on multi-channel RSSI data, the system can effectively distinguish between signal fluctuations due to multipath fading and those resulting from human blockage, leading to significantly improved positioning accuracy. Moreover, the paper [111] showcases the potential of AI in analyzing the mobility and distribution of Bluetooth enabled devices for various applications, including public transportation management and epidemiological investigations. This research demonstrates how AI algorithms can effectively process and interpret BLE data to extract valuable insights into population movements and interactions. The convergence of BLE with AI and ML heralds a future in which IoT networks are not only smarter but also more adaptive, efficient, and capable of autonomous decision-making.

#### 6.1.9. Sustainability and Environmental Impact

Future BLE developments will also focus on sustainability, reducing the environmental impact of IoT devices. This includes designing BLE devices with recyclable materials, improving energy efficiency, and implementing protocols that minimize electronic waste. Sustainable design practices will ensure that the growing number of IoT devices contributes positively to environmental conservation efforts.

#### 6.1.10. Summarize

These emerging trends and advancements in BLE technology will significantly enhance its role in the IoT ecosystem, driving innovation and enabling new applications across various industries. As BLE continues to evolve, it will remain a critical enabler of smart, connected solutions that improve efficiency, convenience, and quality of life.

### 6.2. Research Opportunities: Potential Areas for Further Research and Development

The evolution of BLE technology presents numerous research opportunities that can drive further innovation and enhance its integration into the IoT ecosystem. One key area for research is the development of advanced power management techniques that can further extend the battery life of BLE devices. This includes exploring new energy harvesting methods, such as thermoelectric and piezoelectric energy sources, which can complement or replace traditional batteries. Additionally, there is a need for optimizing communication protocols to minimize power consumption without compromising performance. Another promising research avenue is the enhancement of BLE security features to protect against evolving cyber threats. This involves developing more robust encryption algorithms, secure pairing methods, and intrusion detection systems that can safeguard data integrity and privacy. Furthermore, the integration of quantum-resistant cryptographic techniques could future-proof BLE devices against potential threats posed by quantum computing. Improving the accuracy and reliability of BLE-based location services is also a critical research area. Innovations in direction finding and AoA technologies could enable centimeter-level precision, opening up new applications in indoor navigation, asset tracking, and augmented reality. Research into scalable and efficient mesh networking protocols is essential for supporting large-scale IoT deployments. Developing self-healing and adaptive routing algorithms can enhance network robustness and ensure reliable communication in dynamic environments. The convergence of BLE with other wireless technologies, such as Wi-Fi, cellular, and Long Range (LoRa), presents opportunities for creating hybrid communication solutions that leverage the strengths of multiple standards. Investigating seamless integration techniques and interoperability frameworks will be crucial for developing comprehensive IoT ecosystems. Additionally, the application of AI and ML to optimize BLE communication patterns, predict maintenance needs, and enhance security measures offers significant research potential. Edge computing, where data processing occurs on the device rather than in the cloud, combined with AI and ML, can provide real-time analytics and decision-making capabilities at the network edge. Lastly, there is a growing interest in sustainable and environmentally friendly design practices for BLE devices. Research into biodegradable materials, energy efficient manufacturing processes, and recycling protocols can contribute to reducing the environmental impact of IoT deployments. These research opportunities highlight the vast potential for the further development of BLE technology, ensuring its continued relevance and efficacy in an increasingly connected world.

## 7. Discussion

The discussion presented in this paper has provided a comprehensive overview of Bluetooth Low Energy technology, highlighting its current state, challenges, and future directions. In summary, BLE has emerged as a leading wireless communication standard for Internet of Things applications due to its low power consumption, low cost, and ease of use. However, the technology still faces several challenges, including interoperability issues, security concerns, and limitations in terms of range and coverage. To address these challenges, various solutions have been proposed, such as standardized communication protocols, robust security measures, and advanced power management techniques. Though trade-offs exist, such as balancing power usage with increased transmission ranges or minor delays introduced by security protocols, the versatility and thoughtful design of BLE make it a vital component of today’s IoT ecosystems. Furthermore, emerging trends like LE Audio, AI and ML integration, and sustainability are expected to shape the future of BLE and its applications. The evolution of BLE has significant implications for the broader context of wireless communication and IoT. As the number of connected devices continues to grow, the demand for low-power, low-cost, and reliable wireless communication technologies will increase. BLE is well positioned to meet this demand, enabling a wide range of applications, from smart home automation to industrial control systems. The impact of the evolution of BLE will be felt across various industries, including healthcare, transportation, and manufacturing, where IoT devices are being increasingly adopted. Moreover, the integration of BLE with other wireless technologies, such as Wi-Fi and cellular, will enable seamless communication and create new opportunities for innovative applications. As a result, the significance of the evolution of BLE extends beyond the technology itself, influencing the development of IoT ecosystems and shaping the future of connected devices. In conclusion, the future of BLE looks promising, with ongoing advancements in technology and emerging trends expected to drive its continued evolution. As researchers and developers, it is essential to stay focused on addressing the challenges associated with BLE, while exploring new opportunities for innovation and growth. The integration of AI, ML, and other emerging technologies will play a crucial role in shaping the future of BLE, enabling more efficient, secure, and sustainable wireless communication systems. Ultimately, the evolution of BLE will be shaped by the collective efforts of industry stakeholders, researchers, and developers, working together to create a robust, reliable, and scalable wireless communication ecosystem that supports the growing demands of IoT applications. As we look to the future, it is clear that BLE will remain a vital component of the wireless communication landscape, enabling new possibilities for connected devices and transforming the way we live and work.

## Figures and Tables

**Table 1:** Bluetooth core specification versions.

Bluetooth Version	Release Year	Key Improvements
1.0 [8]	1999	Basic functionality for voice and data transmission over short distances.
1.1 [9]	2001	Improved data transfer rates and error correction.
1.2 [10]	2003	Enhanced security features and support for faster data transfer speeds.
2.0 [11]	2004	Introduced Enhanced Data Rate (EDR) for significantly faster data transfer rates.
2.1 [12]	2007	Improved power management and connection speed.
3.0 [13]	2009	Introduced High Speed (HS) mode for even faster data transfer rates (up to 24 Mbps) and support for new profiles like internet sharing.
4.0 [14]	2010	Introduced BLE for low-power communication.
4.1 [15]	2013	Improvements to BLE functionality and connection establishment.
4.2 [16]	2014	Introduced LE Data Packet Length Extensions for larger data transfers with BLE.
5.0 [17]	2016	Increased BLE data rate (2 Mbps) and improved advertising features.
5.1 [18]	2019	Introduced Direction Finding for improved in-door positioning and GATT improvements.
5.2 [19]	2019	Focused on reliability, power consumption, and coexistence: EATT protocol, dynamic power control, improved signal stability.
5.3 [20]	2021	Enhanced location services (accuracy and privacy), High Data Rate (HDR) LE, Connection Subrating, LE Power Control.
5.4 [21]	2023	Featured advancements in Periodic Advertising with Responses (PAwR), enabling more efficient data transfers and improved location services.
6.0 [22]	2024	Significant enhancements in speed, range, and reliability, with a particular focus on supporting advanced audio applications and IoT devices.

**Table 2:** Comparison of the different classes of Bluetooth devices.

Class	Max Transmission Power	Range	Bluetooth Low Energy	Bluetooth Classic
Class 1	100 mW (+20 dBm)	Up to 100 m (328 feet)	Common in industrial applications, supports long-range communication with low power consumption.	Common in industrial applications, provides robust connectivity for long distances.
Class 1.5 ^1^	10 mW (+10 dBm)	Up to 30 m (98 feet)	Most common in consumer electronics, used in smartphones, wearable devices, and peripherals.	Smart locks, fitness trackers, and wireless audio devices.
Class 2	2.5 mW (+4 dBm)	Up to 10 m (33 feet)	Most common in consumer electronics, used in smartphones, wearable devices, and peripherals.	Common in consumer electronics, used for headsets, keyboards, and mice.
Class 3	1 mW (0 dBm)	Up to 1 m (3 feet)	Less common, used in niche applications requiring short-range communication.	Less common, used in applications with minimal range requirements.
Class 4 ^2^	0.5 mW (−3 dBm)	Up to 0.5 m (1.5 feet)	Rare, hypothetical class for ultra-low power, very short-range applications.	Rare, not commonly used or standardized.

^1^ Class 1.5 is less commonly discussed than the other classes, but it does exist in the Bluetooth specifications. It offers a good compromise for devices that need more range than Class 2 but don’t require the full power of Class 1. ^2^ Class 4 is not a standard classification in the Bluetooth specification but is included here for theoretical completeness in ultra-low power scenarios.

**Table 3:** Technical comparison of Bluetooth Low Energy and Bluetooth Classic.

Feature	Bluetooth Low Energy	Bluetooth Classic
**Channels**	40 channels with 2 MHz spacing	79 channels with 1 MHz spacing
**Throughput**	LE 1M PHY: 1 Mbps LE 2M PHY: 2 Mbps LE 2M 2BT ^1^ PHY: 2 Mbps LE Coded PHY (S = 8): 125 kbps LE Coded PHY (S = 2): 500 kbps	EDR (8DPSK): 3 Mbps EDR (π/4−DQPSK): 2 Mbps Basic Data Rate: 1 Mbps
**Radio Profiles ^2^**	Class 1: 100 mW (+20 dBm) Class 1.5: 10 mW (+10 dbm) Class 2: 2.5 mW (+4 dBm) Class 3: 1 mW (0 dBm)	Class 1: 100 mW (+20 dBm) Class 2: 2.5 mW (+4 dBm) Class 3: 1 mW (0 dBm)
**Power Consumption ^3^**	0.01× to 0.5× of BT Classic	Depends on Radio Profile
**Range ^4^**	Class 1: up to 100 m (328 feet) Class 1.5: up to 30 m (98 feet) Class 2: up to 10 m (33 feet) Class 3: up to 1 m (3 feet)	Class 1: up to 100 m (328 feet) Class 2: up to 10 m (33 feet) Class 3: up to 1 m (3 feet)
**Network Topologies**	P2P Broadcast Mesh	P2P

^1^ The LE 2M 2BT PHY was introduced in Bluetooth Core Specifications 6.0 [22] and it is used exclusively for Channel Sounding. ^2^ Class 1.5 represents an intermediate power class between Class 1 and Class 2. This class was introduced to provide a balance between the longer range of Class 1 and the lower power consumption of Class 2. ^3^ It is worth mentioning that while these classes define the maximum power output, actual devices may operate at lower power levels within their class depending on the specific implementation and power-saving features. ^4^ The actual range of a Bluetooth device depends not just on the transmitter power (which these classes define) but also on the receiver sensitivity and antenna designs of both devices in the connection.

**Table 4:** Key stages in the evolution of BLE.

BLE Version	Release Month/Year	Notable Features and Enchancements
4.0 [14]	6/2010	Introduction of Low-Energy Physical Layer, Low-Energy Link Layer, Enhancements to HCI for Low Energy.
4.1 [15]	12/2013	BR/EDR Secure Connections, Train nudging, Generalized interlaced scan, Low duty cycle directed advertising.
4.2 [16]	12/2014	LE Data Packet Length Extension, LE Secure Connections, Link Layer privacy, Link Layer Extended Scanner Filter policies.
5.0 [17]	12/2016	Slot Availability Mask (SAM), 2 Msym/s PHY for LE, LE Long Range, High Duty Cycle Non-Connectable Advertising, LE Advertising Extensions.
5.1 [18]	01/2019	Angle of Arrival (AoA) and Angle of Departure (AoD), Advertising Channel Index, GATT Caching, Periodic Advertising Sync Transfer.
5.2 [19]	12/2019	LE Isochronous Channels, Enhanced Attribute Protocol, LE Power Control.
5.3 [20]	7/2021	AdvDataInfo in Periodic Advertising, Host to Controller Encryption Key Control Enhancements, LE Enhanced Connection Update, LE Channel Classification.
5.4 [21]	1/2023	Advertising Coding Selection, Encrypted Advertising Data, LE GATT Security Levels Characteristic, Periodic Advertising with Responses.
6.0 [22]	8/2024	Channel Sounding, LL Extended Feature Set, Decision-Based Advertising Filtering, Enhancements for ISOAL, Monitoring Advertisers, Frame Space Update.

## Data Availability

No new data were created or analyzed in this review article; data sharing is not applicable to this study.

## Abbreviations

The following abbreviations are used in this manuscript:

8DPSK	8-phase Differential Phase-Shift Keying
ADAS	Advanced Driver Assistance Systems
AES	Advanced Encryption Standard
AFH	Adaptive Frequency Hopping
AI	Artificial Intelligence
ANNs	Artificial Neural Networks
AoA	Angle of Arrival
AoD	Angle of Departure
AR	Augmented Reality
BLE	Bluetooth Low Energy
Bluetooth SIG	Bluetooth Special Interest Group
BR/EDR	Basic Rate/Enhanced Data Rate
BT	Bandwidth-Bit Period Product
C2C	Car to Car
C2I	Car to Infrastructure
DPSK	Differential Phase-Shift Keying
DQPSK	Differential Quaternary Phase-Shift Keying
EATT	Enhanced Attribute protocol
ECDH	Elliptic Curve Diffie–Hellman
ESL	Electronic Shelf Labels
FEC	Forward Error Correction
FHSS	Frequency Hopping Spread Spectrum
GATT	Generic Attribute profile
GFSK	Gaussian Frequency Shift Keying
GIoT	Green Internet of Things
GIoT-BHMC	Green Internet of Things-Bluetooth Hotmeal Container
HCI	Host Controller Interface
HS	High Speed
IDPS	Intrusion Detection and Prevention Systems
IEEE SA	Institute of Electrical and Electronics Engineers Standards Association
IETF	Internet Engineering Task Force
IIoT	Industrial Internet of Things
IoT	Internet of Things
IPSP	Internet Protocol Support Profile
IRK	Identity Resolving Key
ISM Band	Industrial, Scientific and Medical Band
ISOAL	Isochronous Adaptation Layer
IWSN	Industrial Wireless Sensor Networks
LC3	Low-Complexity Communication Codec
LE Audio	Low-Energy Audio
LoRa	Long Range
LOS	Line of Sight
LPWAN	Low-Power Wide Area Network
LTE	Long-Term Evolution
MAC	Media Access Control
MitM	Man in the Middle
ML	Machine Learning
MTU	Maximum Transmission Unit
OOB	Out of Band
OTA	Over the Air
P2P	Point to Point
PAN	Personal Area Network
PAwR	Periodic Advertising with Responses
PHY	Physical Layer
PKI	Public Key Infrastructure
PSK	Pre-Shared Keys
RF	Radio Frequency
RHMS	Remote Health Monitoring Systems
RPAs	Resolvable Private Addresses
RSSI	Received Signal Strength Indicator
SAM	Slot Availability Mask
SoC	System-on-Chip
STRIDE	Spoofing, Tampering, Repudiation, Information disclosure, Denial of service and
	Elevation of privilege
TPMS	Tire Pressure Monitoring Systems
UUID	Universal Unique Identifier
VR	Virtual Reality
WBMS	Wireless Battery Management Systems
Wi-Fi	Wireless Fidelity
